# Elastic Properties Measurement Using Guided Acoustic Waves

**DOI:** 10.3390/s21196675

**Published:** 2021-10-08

**Authors:** Viktor Fairuschin, Felix Brand, Alexander Backer, Klaus Stefan Drese

**Affiliations:** Institute of Sensor and Actuator Technology (ISAT), Coburg University of Applied Sciences and Arts, Am Hofbräuhaus 1b, 96450 Coburg, Germany; felix.brand@hs-coburg.de (F.B.); alexander.backer@hs-coburg.de (A.B.); klaus.drese@hs-coburg.de (K.S.D.)

**Keywords:** guided acoustic waves, elastic properties, spectral collocation method, Lamb waves

## Abstract

Nondestructive evaluation of elastic properties plays a critical role in condition monitoring of thin structures such as sheets, plates or tubes. Recent research has shown that elastic properties of such structures can be determined with remarkable accuracy by utilizing the dispersive nature of guided acoustic waves propagating in them. However, existing techniques largely require complicated and expensive equipment or involve accurate measurement of an additional quantity, rendering them impractical for industrial use. In this work, we present a new approach that requires only a pair of piezoelectric transducers used to measure the group velocities ratio of fundamental guided wave modes. A numerical model based on the spectral collocation method is used to fit the measured data by solving a bound-constrained nonlinear least squares optimization problem. We verify our approach on both simulated and experimental data and achieve accuracies similar to those reported by other authors. The high accuracy and simple measurement setup of our approach makes it eminently suitable for use in industrial environments.

## 1. Introduction

The field of guided acoustic waves (GAWs) has attracted increasing interest in the research community over the past decade [1]. In addition to various applications in nondestructive testing [2,3] and structural health monitoring [4,5], guided waves have been successfully used to measure liquid properties [6], monitor biogenic deposits in tubes [7], and even to accelerate electrochemical reactions during the battery charging process [8].

Guided acoustic waves are dispersive, i.e., their phase and group velocities depend on frequency [9]. For particular material and geometry, this dependence is usually represented by dispersion curves, which have proven to be a useful tool for the development of GAW applications [10,11]. Consequently, various methods have been developed to compute dispersion curves for different scenarios, including cylindrical [12,13,14] and multilayer systems [15,16,17], as well as waveguides surrounded by liquids [18,19,20].

Using the opposite approach, the dispersive nature of GAWs can be utilized to determine the elastic properties of thin structures such as sheets, plates, and tubes. The procedure first requires the measurement of group or phase velocities at multiple frequencies. A numerical model is then fitted to these measurements by optimizing the model parameters. Recent research in this area has shown that this strategy can determine the elastic properties of thin structures with remarkable accuracy [21,22,23,24,25]. However, existing studies differ widely in terms of how the velocities are measured.

One of the earliest works on this subject was published by Rogers [21] in the mid-nineties. He used a pair of variable-beam contact transducers to measure phase velocities of different Lamb-type modes, by considering the phase shift over a certain path. Sale et al. [22] used a pair of piezoelectric transducers at a fixed distance to measure the group velocities of zero order guided wave modes excited by a laser. In another publication [23] a speckle interferometer was used to measure the wavelength of multiple guided wave modes, excited using a transducer wedge. A similar approach was reported [24], where a laser-scanning vibrometer was used to measure the wavenumbers of different Lamb-type modes. Gao et al. [25] used a laser ultrasonic setup for both, excitation and detection of different Lamb modes.

Most of the described techniques either require complicated and expensive equipment or involve accurate measurement of an additional quantity, rendering them less suitable for use outside the laboratory environment. In this work, we introduce a new simple and precise approach that requires only a pair of piezoelectric transducers used to measure the group velocities ratio of fundamental Lamb modes. A numerical model based on spectral collocation method is used to fit the measured data, and both longitudinal and transverse wave velocities and the thickness of the inspected structure are estimated by solving a bound-constrained nonlinear least squares optimization problem.

## 2. Materials and Methods

In the following section, we introduce the measurement setup, the numerical model used to fit the measured data and the optimization approach used to extract the elastic properties of the inspected structure.

### 2.1. Measurement Setup

**Figure 1:**
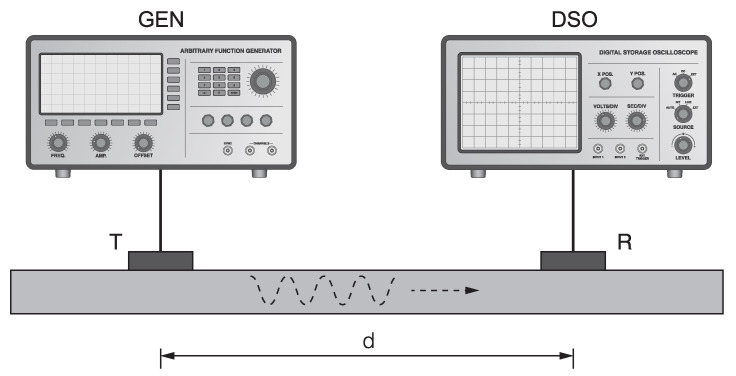
A schematic diagram of the measurement setup, consisting of a signal generator (GEN), a digital storage oscilloscope (DSO) and two transducers, one for transmitting (T) and one for receiving (R) the guided acoustic wave signals, attached to the surface of the test specimen at a fixed distance (d).

The measurement setup involves a pair of identical piezoelectric disc transducers (PIC255, diameter 8 mm, thickness 0.5 mm, PI Ceramic GmbH, Lederhose, Germany), attached to the surface of the test specimen at a fixed distance d= 100 mm, as shown in Figure 1. The transmitting transducer is excited by a signal generator (33500B Series, Keysight Technologies, Inc., Santa Rosa, CA, USA), which provides five-cycle sine bursts modulated by a Hanning window. A digital storage oscilloscope (HDO6034 (Teledyne LeCroy, Chestnut Ridge, NY, USA) is used to record the Lamb-type guided acoustic wave signals arriving at the receiving transducer.

**Figure 2:**
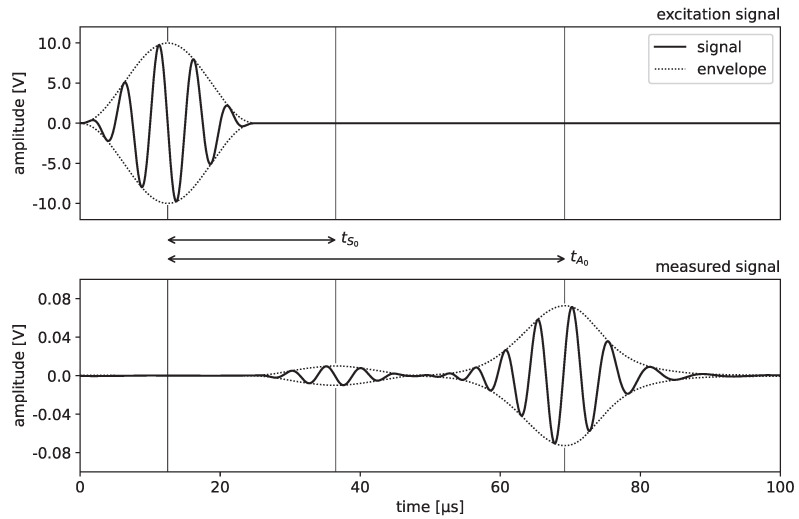
Determination of propagation times of the fundamental Lamb modes.

A typical signal recorded by the oscilloscope is demonstrated in Figure 2. In order to extract the propagation times $t_{A_{0}}$ and $t_{S_{0}}$ of both fundamental Lamb modes, the envelope of the signal is calculated and the time shifts of the maxima relative to the excitation signal are determined. We note that, depending on the application, other approaches such as those based on computation of the wavelet transform [26] or the short-time Fourier transform [27] may also be considered for this purpose.

Once the propagation times have been determined, group velocities $c_{A_{0}}$ and $c_{S_{0}}$ are usually calculated by considering the distance *d* between the transmitter and the receiver

(1) $$c_{A_{0}}=\frac{d}{t_{A_{0}}}\;\;\mathrm{and}\;\;c_{S_{0}}=\frac{d}{t_{S_{0}}}.$$

However, accurate measurement of the distance *d* not only requires additional equipment, but also affects the measurement uncertainty of the group velocities. Our approach overcomes this issue using a simple trick: instead of estimating the velocities directly, we calculate their ratio and thus eliminate the distance *d* in Equation (1), yielding

(2) $$y=\frac{c_{S_{0}}}{c_{A_{0}}}=\frac{t_{A_{0}}}{t_{S_{0}}}.$$

### 2.2. Numerical Model

A popular approach used to calculate dispersion curves of a plane waveguide, is to solve the analytical frequency equations using root-finding techniques [1]. However, for complicated problems the root-finding process becomes increasingly challenging, particularly if multilayer systems need to be examined [28]. For these scenarios, matrix methods are usually preferred.

Matrix methods compose the individual solutions of each layer into a system matrix and the general solution, which satisfies the boundary and the interface conditions, is then calculated for the entire system. This is done by finding the roots of the characteristic function, which is represented by the determinant of the system matrix (a detailed treatment of matrix methods can be found in [15]). However, matrix methods are prone to numerical errors and are usually associated with high computational costs [29].

In this work, we employ an alternative popular method recently introduced by various authors [28,29,30,31,32,33], which allows the dispersion curves to be calculated by numerically solving the underlying differential equations using the spectral collocation method. This method provides a good trade-off between precision, implementation effort, and computation time, and can be easily applied to cylindrical geometries or multilayer systems [28,30]. However, the spectral collocation method is not always robust. A detailed discussion on the robustness of this method in the calculation of dispersion curves of acoustic waveguides has already been provided by other authors (see, e.g., references [29,30,31]). For applications where robustness is critical, the approach may need to be adapted.

#### 2.2.1. Derivation of Differential Equations

**Figure 3:**
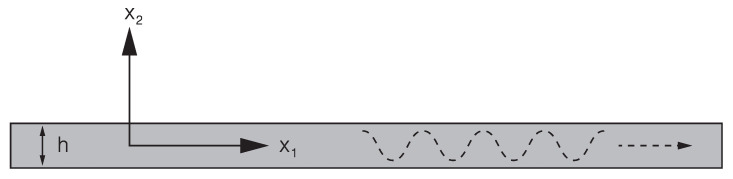
Geometry of the waveguide.

To introduce the numerical model, we consider a planar waveguide as shown in Figure 3. Following [34], we express the displacement field in terms of potentials Φ and Ψ and thus obtain two uncoupled wave equations

(3) $$\begin{array}{cc} \frac{\partial^{2}\Phi }{\partial x_{1}^{2}}+\frac{\partial^{2}\Phi }{\partial x_{2}^{2}}\; & =\frac{1}{c_{L}^{2}}\frac{\partial^{2}\Phi }{\partial t^{2}},\\ \frac{\partial^{2}\Psi }{\partial x_{1}^{2}}+\frac{\partial^{2}\Psi }{\partial x_{2}^{2}}\; & =\frac{1}{c_{T}^{2}}\frac{\partial^{2}\Psi }{\partial t^{2}}, \end{array}$$

for longitudinal and transverse waves, which propagate independently in unbounded solid medium with velocities $c_{L}$ and $c_{T}$, respectively. The coupling of both wave equations is provided by the boundary conditions at the surfaces of the waveguide, requiring both stress components $\sigma_{21}$ and $\sigma_{22}$ to be equal to zero

(4) $$\begin{array}{cc} \sigma_{21} & =\mu \left(\frac{\partial u_{2}}{\partial x_{1}}+\frac{\partial u_{1}}{\partial x_{2}}\right)=0,\\ \sigma_{22} & =\lambda \left(\frac{\partial u_{1}}{\partial x_{1}}+\frac{\partial u_{2}}{\partial x_{2}}\right)+2\mu \frac{\partial u_{2}}{\partial x_{2}}=0, \end{array}$$

where λ and μ are Lamé constants and the displacement components $u_{1}$ and $u_{2}$ are defined as follows

(5) $$\begin{array}{cc} u_{1} & =\frac{\partial \Phi }{\partial x_{1}}+\frac{\partial \Psi }{\partial x_{2}},\\ u_{2} & =\frac{\partial \Phi }{\partial x_{2}}-\frac{\partial \Psi }{\partial x_{1}}. \end{array}$$

For solutions of the wave equations we consider plane harmonic waves of the type

(6) $$\begin{array}{cc} \Phi (x_{1},x_{2},t) & =\phi \left(x_{2}\right)e^{i(kx_{1}-\omega t)},\\ \Psi (x_{1},x_{2},t) & =i\psi \left(x_{2}\right)e^{i(kx_{1}-\omega t)}, \end{array}$$

where *k* is the wavenumber, *t* the time, ω the angular frequency and *i* the imaginary number. By substituting (6) into wave Equation (3) we obtain

(7) $$\begin{array}{cc} \left(\frac{\partial^{2}}{\partial x_{2}^{2}}-k^{2}\right)\phi \; & =-\frac{\omega^{2}}{c_{L}^{2}}\phi ,\\ \left(\frac{\partial^{2}}{\partial x_{2}^{2}}-k^{2}\right)i\psi \; & =-\frac{\omega^{2}}{c_{T}^{2}}i\psi . \end{array}$$

By analogy, substituting (6) into (4) delivers

(8) $$\begin{array}{cc} \frac{i\sigma_{21}}{\mu } & =\left(-2k\frac{\partial }{\partial x_{2}}\right)\phi +\left(\frac{\partial^{2}}{\partial x_{2}^{2}}+k^{2}\right)i\psi =0,\\ \frac{\sigma_{22}}{2\mu } & =\left(\left(\alpha +1\right)\frac{\partial^{2}}{\partial x_{2}^{2}}-\alpha k^{2}\right)\phi -\left(k\frac{\partial }{\partial x_{2}}\right)i\psi =0, \end{array}$$

where

(9) $$\alpha =\frac{\lambda }{2\mu }\equiv \frac{1}{2}\frac{c_{L}^{2}}{c_{T}^{2}}-1.$$

#### 2.2.2. Spectral Collocation Method

The spectral collocation method (SCM) is a numerical approach to solving differential equations. Its main idea is to expand the solution of the differential equation by a linear combination of some global polynomial basis functions that satisfy the exact solution on a set of *N* pre-assigned collocation points. This allows the evaluation of derivatives at such collocation points by multiplication with a pre-computed matrix. For bounded non-periodic domains, Chebyshev differentiation matrices are typically used [35].

Following this scheme, we replace the *m*-th order differential operators in Equation (7) with the corresponding Chebyshev differentiation matrices $D^{\left(m\right)}$ (see, e.g., [36] for implementation details) and thus obtain a system of equations

(10) $$\left[L\right]\left(\begin{array}{c} \phi \\ i\psi \end{array}\right)=\omega^{2}\left[M\right]\left(\begin{array}{c} \phi \\ i\psi \end{array}\right),$$

where

(11) $$\left[L\right]=\left[\begin{array}{cc} D^{\left(2\right)}-k^{2}\;\cdot \;I & 0\\ 0 & D^{\left(2\right)}-k^{2}\;\cdot \;I \end{array}\right],$$

(12) $$\left[M\right]=\left[\begin{array}{cc} -1/c_{L}^{2}\;\cdot \;I & 0\\ 0 & -1/c_{T}^{2}\;\cdot \;I \end{array}\right]$$

and *I* is an N×N identity matrix.

Similarly, from Equation (8) it follows that

(13) $$\left[S\right]\left(\begin{array}{c} \phi \\ i\psi \end{array}\right)=0,$$

where

(14) $$\left[S\right]=\left[\begin{array}{cc} -2kD^{\left(1\right)} & D^{\left(2\right)}+k^{2}\;\cdot \;I\\ \left(\alpha +1\right)D^{\left(2\right)}-\alpha k^{2}\;\cdot \;I\; & -kD^{\left(1\right)} \end{array}\right].$$

At this point, the wave equations and boundary conditions are both represented by a set of 2N equations corresponding to *N* Chebyshev collocation points defined on the interval [−1,1]. Using coordinate transformation

(15) $$D_{\left[-\frac{h}{2},\frac{h}{2}\right]}^{\left(m\right)}={\left(\frac{2}{h}\right)}^{m}D_{\left[-1,1\right]}^{\left(m\right)},$$

the solution is introduced on the problem domain.

Finally, (10) and (13) are combined into a system of equations by replacing the rows 1, *N*, N+1 and 2N of the matrix [L] with the corresponding rows of the matrix [S] and setting these rows to zero in the matrix [M].

Once the eigenvalues $\omega^{2}$ are calculated for different *k* values by solving the matrix eigenvalue problem (10) using the QZ algorithm [37], the group velocities are calculated as follows

(16) $$c=\frac{d\omega }{dk}.$$

The above equations combine to give the numerical model M, which is used to fit the measured data, as described in the next section.

### 2.3. Optimization

In our approach, we use the numerical model described in the previous section to fit our measured data. The elastic properties and thickness of the test specimen are determined by solving an optimization problem that can be formulated as follows: Consider *n* measurements $y_{i}$ acquired at frequencies $f_{i}$ such that $f_{i}\neq f_{j}\;\forall i\neq j$ and i,j∈{1,2,⋯,n}. We wish to describe the relationship between the frequency *f* and the measured quantity *y* using a numerical model M(f;ϑ) whose parameters form vector $\vartheta ={\left(c_{L},c_{T},h\right)}^{T}$. As a measure for the discrepancy between our model and the observed data, we define the residuals

(17) $$r_{i}\left(\vartheta \right)=y_{i}-M\left(f_{i};\vartheta \right)$$

and finally obtain a nonlinear least squares problem

(18) $$\begin{array}{cc} \; & \min_{\vartheta }\sum_{i=1}^{n}r_{i}^{2}\left(\vartheta \right), \end{array}$$

which can be solved using standard techniques such as the Levenberg–Marquardt algorithm [38].

From a practical point of view, it may be useful to define a search region for the model parameters to prevent the algorithm from getting stuck in a local optimum. In such cases, the task is transformed into a bound-constrained optimization problem, which can be solved efficiently using the STIR (Subspace Trust Region Interior Reflective) method [39].

## 3. Results

### 3.1. Simulated Data

To verify our approach, we first applied it to simulated data. Guided acoustic wave signals in the frequency range 110 to 200 kHz were provided by a finite element model of an h= 1 mm thick aluminium sheet. The elastic properties of aluminium ($c_{L}=$ 6.35 km/s, $c_{T}=$ 3.10 km/s, ρ= 2.8 $g/cm^{3}$) were chosen according to the literature [1]. According to Adamou [30], the number of collocation points *N* required to determine the group velocities of *j* lowest Lamb modes with an accuracy of 10 digits can be calculated with the formula N≥2j+10. For our numerical model, we used a grid of N= 16 collocation points, which proved to be a good trade-off between accuracy and computation time. The parameter bounds were chosen as follows: 5.00 km/s $\leq c_{L}\leq$ 7.00 km/s, 2.50 km/s $\leq c_{T}\leq$ 3.50 km/s, and 0.90 mm ≤d≤ 1.10 mm.

**Figure 4:**
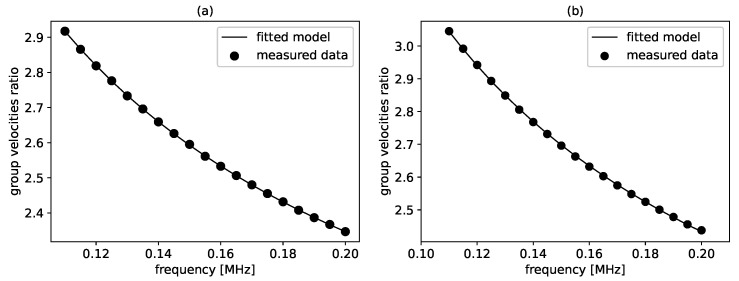
Group velocities ratios (circles) derived from the acoustic signals at different frequencies and the corresponding fitted model (solid line) for (**a**) simulated data and (**b**) experimental data.

The group velocities ratios derived from the simulated acoustic signals at different frequencies as well as the corresponding solution found by optimization are shown in Figure 4. The good agreement between the data and the solution indicates that the algorithm was able to find an optimum. In fact, the relative error between the determined parameters and the reference was about 1%, as can be seen in Table 1.

Since optimization results typically depend on the initial solution, or in our case on the search region specified for the solution, we repeated the optimization process multiple times with some randomly chosen upper and lower parameter bounds. In each case, the relative error between the determined parameters and the reference was about 1%.

### 3.2. Experimental Data

We also applied our approach to experimental data. For this purpose, an aluminium sheet of h=1 mm thickness was investigated. A pair of piezoelectric disc transducers were attached to its surface as described in Section 2.1. The excitation signals in the frequency range 110 to 200 kHz were provided by a signal generator and the received signals were recorded using a digital storage oscilloscope. For the numerical model we used a grid of N= 16 collocation points and the search region for the solution was set in the same way as it was for the simulated data.

To verify the experimental results, additional reference measurements were performed. The thickness of the aluminium sheet was measured using a micrometer screw gauge and the longitudinal wave velocity was obtained by time-of-flight measurements with an ultrasonic transducer (M2017 Delay Line Transducer, Olympus Europa SE & Co. KG, Hamburg, Germany) operating at 30 MHz.

The group velocities ratios obtained from the measured acoustic signals and the corresponding solution are shown in Figure 4. Compared to the simulated data, some measurement points deviate slightly from the solution found. However, the obtained parameters still show good agreement with the reference, as can be seen in Table 2. Although the relative error is slightly higher in this case, the result can still be termed accurate.

## 4. Discussion

This paper presents a method for measuring the elastic properties of thin structures such as tubes, plates and sheets, that takes advantage of the dispersive nature of guided acoustic waves. The measurement setup consists of a signal generator, a digital storage oscilloscope and a pair of piezoelectric transducers, which are used to measure the ratio of group velocities at different frequencies. The measured data is then fed into a numerical model based on the spectral collocation method in order to determine the elastic properties and the thickness of the investigated structure via optimization. Validation of the method with simulated and experimental data resulted in a measurement error of 1.1% and 3.4%, respectively, which is similar to the results reported by other authors [21,22,23,24,25].

To determine the wave velocities from the signals measured with a piezoelectric receiver, the distance between the transmitter and the receiver must be known exactly. This requires additional equipment, complicates the measurement setup and raises the possibility of additional measurement uncertainties, which may have a negative impact on the accuracy of the measurement principle. Our approach overcomes this issue by determining the ratio of the group velocities, which can be calculated directly from the propagation times of the two fundamental Lamb modes, significantly simplifying the entire measurement setup.

Laser ultrasonic methods for detection of guided acoustic waves usually rely on the measurement of the wave number rather than the wave velocity. This also avoids the need to determine the distance between the transmitter and receiver. However, such methods require expensive and complicated equipment and are poorly suited for the characterisation of curved or weakly reflective materials. Our approach, on the other hand, uses low-cost components and allows the characterisation of arbitrarily shaped structures and various materials, opening up a wide field of potential applications.

For the determination of the propagation velocity of the two fundamental Lamb modes, a five-cycle sine burst modulated by a Hanning window is used. On the one hand, the propagation time of the wave can be determined relatively accurately using such a compact signal. On the other hand, such a signal has a much broader spectrum than signals with a higher number of cycles, which means that unwanted side effects such as dispersion become more prominent. In the application studied in this article, the Lamb waves are excited at frequencies with a relatively flat course of the frequency-dependent phase velocity, so that dispersion effects only play a minor role. The relatively small distance between transmitter and receiver also has a positive impact. However, depending on the application, such effects can have a negative impact on the measurement method, which is why the use of alternative signal patterns should be investigated in the future.

For future investigations, the method could be adapted for rotationally symmetrical structures such as pipes. In this case, the measurement setup could be reduced to a single transducer that can act as both a transmitter and a receiver of circumferential Lamb waves. In this way, the measurement of wear or deposits in pipes could be provided, enabling continuous online monitoring of industrial plants. However, in contrast to many non-destructive tests performed on pipes and sheet material, the approach here is not aimed at detecting defects but at determining material properties. A defect-free part of the structure must always be selected so that the measurements are not affected by these non-representative inhomogeneities. In this context, weld metal must also be considered a defect, as the homogeneity of the material is no longer given and the evaluation of the measurement results can lead to misleading values.

## Figures and Tables

**Table 1:** Results for simulated data.

Parameter	Reference	Solution	Error (%)
$c_{L}$ (m/s)	6350.00	6418.44	1.08
$c_{T}$ (m/s)	3100.00	3121.98	0.71
*h* ()	1.00	1.01	0.90

**Table 2:** Results for experimental data.

Parameter	Reference	Solution	Error (%)
$c_{L}$ (m/s)	6718.75	6876.13	2.34
$c_{T}$ (m/s)	-	3151.11	-
*h* (mm)	1.00	0.97	3.40

## Data Availability

Not applicable.
